# Evaluating nanoparticle localisation in glioblastoma multicellular tumour spheroids by surface enhanced Raman scattering†

**DOI:** 10.1039/d3an00751k

**Published:** 2023-06-16

**Authors:** Samantha M. McCabe, Gregory Q. Wallace, Sian Sloan-Dennison, William J. Tipping, Neil C. Shand, Duncan Graham, Marie Boyd, Karen Faulds

**Affiliations:** a Department of Pure and Applied Chemistry, Technology and Innovation Centre, University of Strathclyde 99 George Street Glasgow G1 1RD UK karen.faulds@strath.ac.uk; b The Defence Science and Technology Laboratory (Dstl) Porton Down Salisbury SP4 0JQ UK; c Strathclyde Institute of Pharmacy and Biomedical Sciences, University of Strathclyde 161 Cathedral Street Glasgow G4 0RE UK

## Abstract

Glioblastoma multiforme (GBM) is a particularly aggressive and high-grade brain cancer, with poor prognosis and life expectancy, in urgent need of novel therapies. These severe outcomes are compounded by the difficulty in distinguishing between cancerous and non-cancerous tissues using conventional imaging techniques. Metallic nanoparticles (NPs) are advantageous due to their diverse optical and physical properties, such as their targeting and imaging potential. In this work, the uptake, distribution, and location of silica coated gold nanoparticles (AuNP-SHINs) within multicellular tumour spheroids (MTS) derived from U87-MG glioblastoma cells was investigated by surface enhanced Raman scattering (SERS) optical mapping. MTS are three-dimensional *in vitro* tumour mimics that represent a tumour *in vivo* much more closely than that of a two-dimensional cell culture. By using AuNP-SHIN nanotags, it is possible to readily functionalise the inner gold surface with a Raman reporter, and the outer silica surface with an antibody for tumour specific targeting. The nanotags were designed to target the biomarker tenascin-C overexpressed in U87-MG glioblastoma cells. Immunochemistry indicated that tenascin-C was upregulated within the core of the MTS, however limitations such as NP size, quiescence, and hypoxia, restricted the penetration of the nanotags to the core and they remained in the outer proliferating cells of the spheroids. Previous examples of MTS studies using SERS demonstrated the incubation of NPs on a 2D monolayer of cells, with the subsequent formation of the MTS from these pre-incubated cells. Here, we focus on the localisation of the NPs after incubation into pre-formed MTS to establish a better understanding of targeting and NP uptake. Therefore, this work highlights the importance for the investigation and translation of NP uptake into these 3D *in vitro* models.

## Abstract 2

Combining SERS and immunohistochemistry provides greater insight into how AuNPs distribute in *in vitro* 3D MTS models.

## Graphical abstract

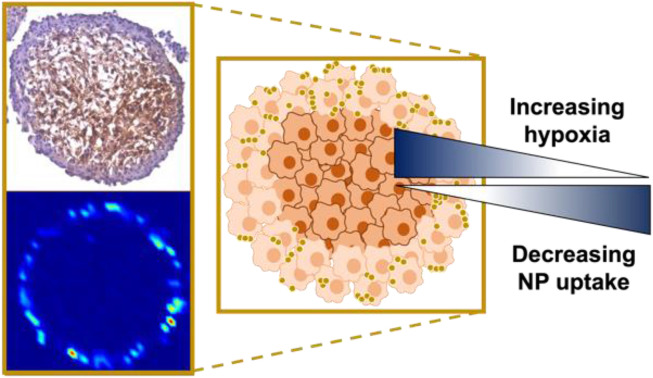

## Introduction

With nearly 400 000 new cases of cancer identified in the UK each year,^1^ it has become increasingly important to develop new methods for detecting cancer. This is especially true for early stages of many cancers, including brain cancer, where the patients’ prognosis and survival can decrease dramatically with late diagnosis.^2^ In this regard, moving away from classical invasive procedures such as biopsies, to faster, non-invasive methods is required, whether for detection, diagnosis, or treatment. *In vitro* translational cancer research typically uses two-dimensional (2D) cell monolayers grown in cell culture. Although this provides crucial information regarding cellular interactions, phenotypical markers and notably membrane-bound proteins, these systems are best described as single-cell experiments. As such, 2D cell monolayers are not a true representation of tumours *in vivo* as the cells are homogeneous and lack tumour heterogeneity with respect to growth rates, and oxygen and nutrient gradients. Additionally, there is an absence of cell-to-cell interactions which do not replicate the true cellular environment within a tumour which is better represented in *in vivo* models.^3,4^ Multicellular tumour spheroids (MTS) are three-dimensional (3D) collections of cells that better represent an *in vivo* environment and act as models for tumours without the need for an animal host. An important aspect of MTS is the formation of an everchanging microenvironment. Within tumours there is a highly proliferative portion of actively replicating cells usually located in close proximity to blood vessels.^5^ However, as the number of cells increases, those that are closer to the core, and hence further from the available blood flow, become quiescent then hypoxic and necrotic due to the reduction in oxygen and nutrient availability.^6^ Specifically, MTS with a size of approximately 200–300 μm will express a hypoxic core and those of 500 μm will have a necrotic core.^7^ Although MTS are not an exact mimic of an *in vivo* tumour, which is vascularised and contains many other cell types and characteristics, they are simply offered as an interim step between 2D cellular monolayers and *in vivo* models as they do present many similarities mentioned. Since 2D cellular monolayers lack these commonalities with *in vivo* tumours, the progression of drug and disease therapies translated from 2D cultures to *in vivo* often fails.^8^ Therefore MTS better replicate this microenvironment and act as an intermediate between 2D culture and *in vivo* models. Furthermore, this intermediate step could result in the use of less animal models being used, which is beneficial both financially and economically. For example, MTS can be used to study the uptake of species, specifically metallic nanoparticles (NPs), by the enhanced permeability and retention (EPR) effect. In this phenomenon, molecules and nanomaterials are retained within the tumour due to the more leaky endothelial vasculature and poor lymphatic drainage of rapidly growing cancer cells over healthy cells.^9^

Nanoparticles, in particular gold nanoparticles (AuNPs) have been used extensively in cancer research due to their tuneable size, surface chemistry, biocompatibility and targeting abilities.^10,11^ In terms of cellular uptake, the size of the AuNPs and the cell line are both important aspects that need to be considered.^11,12^ In a critical comparison between MCF-7 breast cancer cells in a 2D monolayer, *ex vivo* MTS and *in vivo* mice models,^13^ it was found that the depth penetration of the AuNPs was reduced significantly due to the tumour microenvironment in the MTS and *in vivo* studies. This demonstrates the importance of moving from 2D cell monolayers to 3D cultures that more closely represent the *in vivo* environment. In this regard, 3D MTS have emerged as an attractive option for investigating cellular uptake as it represents a logical progression towards the clinic compared to 2D cell monolayers.^14–17^

In addition to NP size, it is essential to consider how to optimise targeting for potential cellular translation. The extracellular matrix (ECM) offers opportunities for targeted delivery of NPs by conjugating the outer NP surface with a specific biomolecule (*i.e.*, an antibody or peptide). For example, tenascin-C is a glycoprotein that is expressed in the ECM of tissues during disease or injury,^18^ and it is overexpressed in many cancer cell lines.^19^ It can bind to cell surface receptors, such as toll-like receptor 4 (TLR4), which is present on stromal cells.^20^ Furthermore, tenascin-C has been found in the stroma of solid tumours^21^ and importantly, glioblastoma cells, such as U87-MG cells, are known to express high levels of tenascin-C.^22,23^ By conjugating a peptide that can interact with tenascin-C to the surface of silver NPs, it was found that the NPs with the peptide were up taken by the U87 cells, while no uptake was observed with NPs lacking the peptide.^24^ Similarly, functionalising antibodies to NPs for a chosen biomarker has been demonstrated in MCF-7 breast cancer MTS, as a potential targeting moiety.^25^ Therefore, there has been a significant body of research based on NP uptake in cells for 2D monolayers and 3D spheroids. However, there is still a lot of work to be done to fully understand the internalisation of different AuNPs into different cancer cell lines for diagnostics, detection, and treatment. The previous research showed that uptake can vary significantly between NP type and size as well as cell type and that 2D culture is not always representative of the 3D environment.

Evaluating the uptake of NPs into cells requires a means of detecting the presence of the NPs in the biological system. Techniques such as scanning electron and transmission electron microscopies can provide the spatial distribution of NPs within the sample but are destructive. With a desire to utilise non-destructive and clinically relevant methods, an alternative approach is required. Surface enhanced Raman scattering (SERS) has emerged as a powerful analytical technique, particularly in cancer diagnostics.^10,25^ By functionalising the metallic NPs with a Raman reporter, it is possible to readily track the distribution of the NPs within a sample based on the unique fingerprint of the reporter.^26,27^ In this regard, SERS offers high sensitivity and multiplexing capabilities.^28,29^ A method previously used for detection of NPs using SERS is the incubation of the AuNPs on a 2D monolayer of cells, with the later formation of the MTS.^30,31^ Although as a first port of call this is useful to observe information regarding the NPs within 3D MTS, and whether they could be detected, it does not provide a true representation of the accumulation of NPs within a 3D MTS as a step towards understanding potential NP uptake into tumours *in vivo*. In fact, it bypasses many of the possible uptake obstacles the NPs would encounter within a tumour. The progression from 2D *in vitro* studies to *in vivo* animal studies is often difficult to reproduce and therefore investigation of 3D *in vitro* models is a critical aspect for understanding the uptake and localisation of NPs within a tumour after it has formed, providing an approach more representative of tumour targeting *in vivo*.

In this work, we reveal the importance of 3D *in vitro* studies as a bridge in cancer research between 2D *in vitro* and *in vivo* animal models through investigation of the localisation of targeted shell isolated AuNPs (AuNP-SHINs) in glioblastoma MTS using SERS. Immunohistochemistry was used to observe the cellular morphology and further assess NP uptake in the MTS.

## Experimental

### Materials

All materials used were purchased from Sigma-Aldrich Ltd (Dorset, UK), unless stated otherwise. The U87-MG glioblastoma cancer cell line was purchased from Caliper Life Sciences (Waltham, Massachusetts, US). Eagle's minimum essential medium (EMEM), Foetal Bovine Serum (FBS), Amphotericin-B, Penicillin/Streptomycin, Histoplast, pelletised Paraffin Wax and DPX mounting medium were purchased from Thermo Fisher Scientific (Leicestershire, UK). Histoclear clearing agent was purchased from National Diagnostics (Hull, UK). Rabbit monoclonal antibody to tenascin-C (ab108930), goat anti-rabbit IgG (ab6702), goat anti-rabbit IgG (Alexa 488) (ab150077), goat anti-rabbit horse radish peroxidase (HRP) conjugated antibody (ab6721), mounting media with DAPI (ab104139) and DAB substrate kit (ab64238) were purchased from Abcam (Cambridge, UK). Hydroxyprobe Plus Kit including the pimonizadole, primary and secondary antibodies was bought from Hydroxyprobe.com. All glassware was decontaminated with aqua regia prior to use (3 : 1, HCl : HNO_3_). *Caution: aqua regia is highly corrosive and must be handled with caution.*

### Cell culture

U87-MG glioblastoma cancer cells were cultured in Eagle's minimum essential medium (EMEM) containing 0.02 mM phenol red and 2 mM l-glutamine supplemented with 10% heat-inactivated foetal bovine serum (FBS), 100 μg mL^−1^ penicillin/streptomycin and 2 μg mL^−1^ amphotericin B. Cells were incubated at 37 °C and 5% CO_2_ in a humidified incubator. The cells were grown in T75 cell culture flasks and those with a confluence of *ca.* 70–80% were detached using 0.05% trypsin-EDTA and re-suspended in media and counted using a haemocytometer before reseeding or using in experiments.

### Multicellular tumour spheroid (MTS) culture

For the formation of the MTS, U87-MG cells were harvested and counted, and 2.5 × 10^6^ cells were added to a Techne spinner flask with 75 mL of complete (with supplements) EMEM medium, including 10% heat-inactivated foetal bovine serum (FBS), 100 μg mL^−1^ penicillin/streptomycin and 2 μg mL^−1^ amphotericin B. The flasks were gassed with CO_2_ and left to stir in a Techne stirrer flask for 4 days in an incubator set to 37 °C, with regular media changes and addition of CO_2_ every two days.

### Nanoparticle incubation

Details regarding the synthesis and characterisation of the nanoparticles and nanotags are provided in the ESI.† For single cell analysis, the U87-MG glioblastoma cells were seeded and left to grow for 48 hours in a tissue culture 6-well dish. After 48 hours, they were incubated with the Ab nanotags and cAb nanotags for 4 hours at 37 °C and 5% CO_2_. After incubation, the nanotag solution was removed and the cells were washed with phosphate buffered saline (PBS), then fixed using 4% paraformaldehyde (PFA) for 15 minutes at room temperature (RT). They then were washed with PBS and stored at 4 °C prior to mapping.

U87-MG MTS were formed as described, and the spheroids were collected in 1 mL of fresh medium in a bijou container. The Ab and cAb nanotags were added to the MTS at the desired concentration for the study (32 pM or 128 pM) and incubated for the desired time (2, 4, 6, 8 or 24 hours) on a roller to allow for cell uptake into the spheroids, and to prevent the spheroids clumping within the bijou.

### MTS sectioning

The MTS were sectioned using a microtome (LEICA, RM2125RTF). Firstly, the spheroids were fixed in 4% PFA for 1 hour at RT. They were then washed in PBS and added to a bio-wrap (Leica Biosystems Richmond, USA) and placed in a plastic cassette. The cassette was then placed into increasing concentrations (70%, 90% and 100%) of ethanol for 1 hour each, then into histoclear for 1 hour. The fixed spheroids were then placed into paraffin wax at approximately 60 °C for 6 hours to allow the wax to penetrate the spheroids. After 6 hours, the spheroids in wax were placed into a mould and allowed to cool to RT and placed at −20 °C for 1 hour before being sectioned. The sections were added to polysine adhesion glass slides (Epredia, Fisher Scientific, USA) and baked at 60 °C for 2 hours for drying.

### Staining

Prior to staining, all spheroid sections were added to histoclear to remove the paraffin wax then rehydrated in decreasing concentrations of ethanol (100%, 90% and 70%) for 2 minutes each. The following staining procedures were used: haematoxylin and eosin (H&E) for nucleus and cytoplasm detection, immunohistochemistry, and immunofluorescence for detection of the tenascin-C targeting antibody and detection of hypoxia using pimonidazole hydrochloride. Details regarding the specific protocols for each method are provided in the ESI.†

### SERS imaging

SERS images were taken using an InVia Raman instrument with 785 nm laser excitation for nanotag signal and 532 nm laser excitation for cellular stretches, using 100% at the sample (785 nm, 20 mW and 532 nm, 30 mW) and 1 second integration time. The images were taken using a 63× NA 1.20 HC PL water immersion objective lens. SERS images were carried out by taking a series of 2D maps to create a simpler 3D reconstruction. 2D XZ SERS maps were taken at various *y* positions in 50 μm increments. The XZ depth profile maps were performed with resolutions of *x* = 10 μm, and *z* = 50 μm. A series of higher *z* resolution (1 μm) 3D maps were taken throughout the entire depth of the MTS by creating a grid of 100 μm using *x* = 10 μm, and *y* = 10 μm in the centre of the spheroid. Details for data processing are provided in the ESI.†

## Results and discussion

Shell isolated AuNPs (AuNP-SHINs) were prepared by carrying out controlled aggregation of AuNPs making them an attractive structure for SERS-based imaging. The AuNPs are isolated within a silica shell which minimises further aggregation when added to cells and cell media.^32,33^ The NPs were synthesised using a seed-mediated process where a modified Turkevich method was used to make the 50 nm AuNP seed.^34^ The Raman reporter, 4-(1*H*-pyrazol-4-yl) pyridine (PPY) was added to the surface of the AuNP seed and encapsulated inside a silica shell made up of sodium silicate and (3-aminopropyl)trimethoxysilane (APTMS).^32^ PPY is a commonly used non-resonant Raman reporter molecule producing a strong SERS spectrum with an intense peak at 956 cm^−1^ (Fig. S1†).

To investigate potential targeting, a tenascin-C antibody (Ab) was immobilised on the surface of the PPY-AuNP-SHIN nanotags by passive adsorption. The Ab conjugation was achieved by adjusting the NP suspension to a pH of 9. Bovine serum albumin (BSA) was added to the surface after antibody conjugation to block any free sites and prevent non-specific binding of the nanotags. PPY-AuNP-SHIN nanotags with a non-specific, control antibody (cAb) that would not interact with tenascin-C in the cells, were also prepared to investigate the uptake of the non-targeted NPs by the MTS. The cAb nanotags were chosen as the control to use in this work because these nanotags best represented the tenascin-C functionalised (Ab) nanotags for comparison. The cAb nanotags were created in the same way as the Ab nanotags, *i.e.*, with the addition of both an antibody and BSA. To confirm the presence of the antibody on the surface of the nanotags, extinction spectroscopy (Fig. S2A†), dynamic light scattering (DLS) and change in zeta potential (Table S1†) were performed. A lateral flow immunoassay (Fig. S2B†) was also carried out to further confirm the successful antibody conjugation and presence of the Ab and cAb at the surface of the nanotags.

The U87-MG glioblastoma cell line was chosen for this work as it overexpresses the tenascin-C targeting protein.^23^ Single cell studies were carried out to ensure that the nanotags were being up taken into the U87-MG cancer cells that were used to grow the MTS (Fig. S3†). This showed that both the Ab and cAb nanotags were up taken into the single cells on three different *Z* planes throughout the cell. Next, the MTS were formed using a spinner flask method, as described in the Experimental section, and had final sizes of approximately 300–400 μm. The nanotags were added to the formed MTS such that the final concentration of nanotags was 32 pM (unless otherwise stated). The MTS and nanotag solution were incubated at 37 °C with 5% CO_2_ for 4 hours on a rotating platform to allow the MTS to be constantly submerged within the nanotags and media mixture during incubation. A distinct colour change (colourless to purple) was observed in the MTS after incubation with the nanotags (Fig. S4A†). After nanotag incubation, the MTS were washed in PBS, fixed in 4% PFA for 1 hour at room RT, then stored in PBS for mapping. A reconstructed 3D SERS image of the MTS was produced to ascertain the AuNP-SHIN nanotag localisation throughout the depth of the MTS. Subsequent cross-sectioning and histopathological staining of the MTS was also carried out.

### Raman and SERS microscopy of MTS treated with Ab and cAb nanotags

**Fig. 1:**
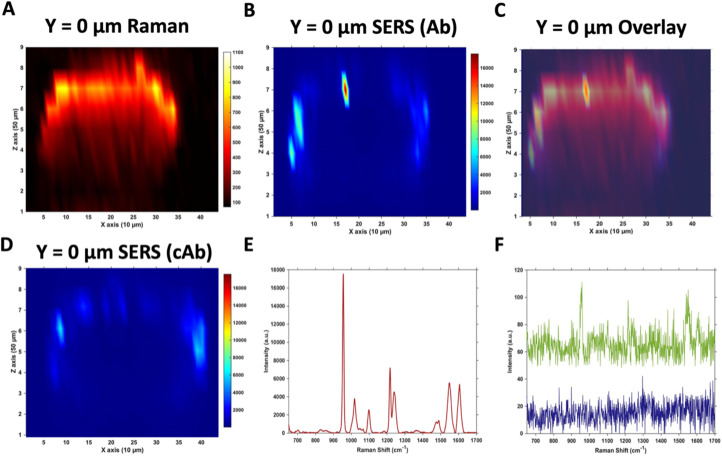
Raman and SERS measurements of U87-MG GBM MTS incubated with 32 pM Ab and cAb nanotags for 4 hours. (A) 2D XZ Raman intensity map at 2930 cm^−1^ showing the CH_3_ cellular protein peak obtained using 532 nm laser excitation taken at *y* = 0 μm of the MTS. (B) 2D XZ SERS intensity maps at 956 cm^−1^ corresponding to the main intensity peak of the Raman reporter PPY on the Ab nanotags obtained using 785 nm laser excitation taken at *y* = 0 μm of the MTS. (C) Overlay of (A) and (B) to illustrate the nanotag signal is within the outer layer of cells of the MTS. (D) 2D XZ SERS intensity maps at 956 cm^−1^ corresponding to the main intensity peak of the Raman reporter PPY on the cAb nanotags obtained using 785 nm laser excitation taken at *y* = 0 μm of the MTS. (E) SERS spectrum of the Ab nanotags at the most intense area of (B) and (F) SERS spectra of the Ab nanotags from the centre of (B) (green) and from a point on the coverslip away from the spheroid (blue). All spectra were taken using a 1 s integration time and 100% laser power (785 nm, 20 mW and 532 nm, 30 mW). All measurements were taken on *n* = 3 MTS.

Due to the large size of the MTS, it was impractical in terms of imaging time to perform high resolution 3D SERS maps of the entire MTS volume. Instead, a series of 2D maps were taken to create a simpler reconstruction (Fig. 1). Here, XZ maps were taken of sections through the MTS at various *y* positions in 50 μm increments. The XZ depth profile maps were performed with resolutions of *x* = 10 μm, and *z* = 50 μm. Label free Raman imaging of the MTS was achieved by reconstructing the intensity of the 2930 cm^−1^ CH_3_ symmetric protein stretch of the cells^35^ to verify that the MTS had cells distributed throughout its entirety and it was not hollow. A 2D XZ Raman map was taken using 532 nm laser excitation at the middle *y* depth (*y* = 0) of the MTS which clearly shows the presence of the 2930 cm^−1^ peak throughout (Fig. 1A). It should be noted that the Raman signal is weaker at the bottom of the MTS as would be expected due to the laser penetrating through a greater depth of material, and the scattered photons having to travel back through the MTS to be collected. A 2D XZ SERS map was collected at the same *y* position using 785 nm laser excitation for nanotag detection. After baseline correction, the intensity of the PPY peak at 956 cm^−1^ was used to generate a false colour SERS intensity map of the Ab nanotags (Fig. 1B). The bright areas of the SERS maps indicate where the nanotags reside within the MTS. By overlaying the two maps, as shown in Fig. 1C, it is possible to correlate the position of strong SERS intensity with the Raman response from the MTS. From this image, we can conclude that there is a clear discrimination observed between the areas where the nanotags were located and where they were absent. It was observed that the Ab nanotags appeared to be located within the outer, spheroid layer which comprises the proliferating cells,^5,36^ and not in the middle of the MTS. A similar observation was found when the cAb was conjugated to the nanotags (Fig. 1D). Examining individual SERS spectra taken from Fig. 1B, a representative spectrum from the outer proliferating layer (Fig. 1E), one from the middle of the MTS (green), and one from the coverslip away from the MTS (blue) (Fig. 1F) show the localisation of the nanotags throughout different areas of the MTS. Corroborating the mapping results, the spectrum taken from the outer layers of the MTS is almost 2 orders of magnitude stronger than the spectrum taken from the middle of the MTS (Fig. 1E and F). Importantly, the XY Raman map in Fig. S5† indicated that the MTS is not hollow. Therefore, while it is possible that some nanotags are within the middle of the MTS, we hypothesise that the weak SERS intensity in the middle of the MTS is the result of a contribution from the much stronger SERS response from the outer layers of the spheroid.

Both sets of nanotags showed a similar distribution in the SERS maps and by plotting them on the same scale it allowed for a comparison in the distribution (Fig. S6†), with the PPY SERS signal being strongest in the outer layer of the MTS. From this experiment, it would suggest that the addition of the tenascin-C targeting antibody onto the surface of the nanotags did not increase the uptake of the nanotags into the middle of the MTS since the uptake of both sets of nanotags appears to be similar (Fig. S6†). Various uptake studies were performed by changing the incubation time and concentration of the nanotags to determine if this would have an impact on deeper penetration of the nanotags into the MTS (Fig. S7†). It was hypothesised that by increasing the incubation time and the concentration, that the nanotags would reach the inner core of the MTS, however, as the cumulative results of Fig. S7† indicate, this was not the case and further investigation was required. It is vitally important to understand nanotag uptake and location of nanotags into MTS to allow for successful translation into *in vivo* studies.

### Understanding the distribution of nanotags in MTS

**Fig. 2:**
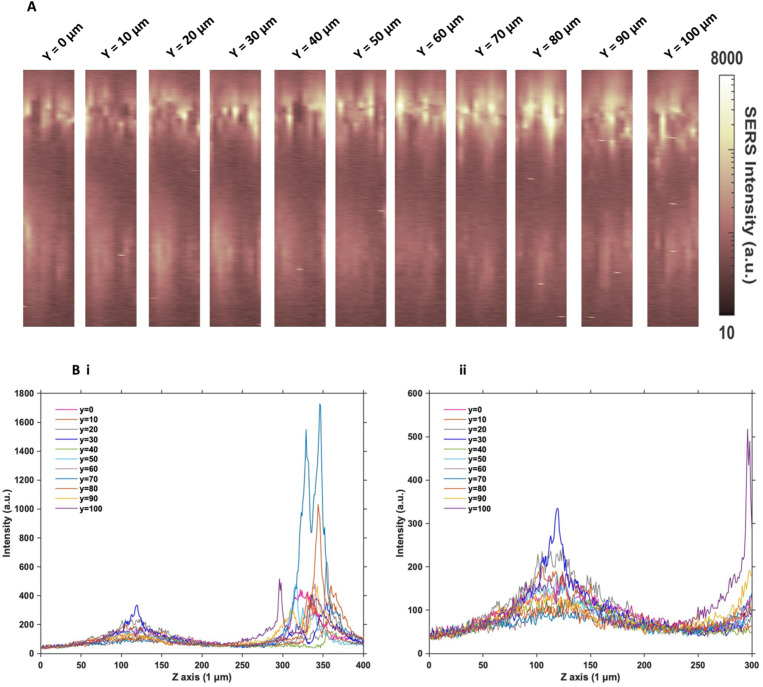
Z optical sectioning through U87-MG GBM MTS incubated with 32 pM Ab nanotags for 4 hours. (A) 3D SERS intensity maps at 956 cm^−1^ corresponding to the intensity of the main peak of the Raman reporter PPY on the nanotags. From left to right illustrates an increasing *y* plane taken throughout the entirety of the MTS at 10 μm increments. (B) Z profile for the PPY intensity illustrating the localisation of the Ab nanotags throughout the entire Z profile of the MTS where (i) shows the full *Z* range and (ii) is an expanded spectrum of the 0 to 300 μm region to show the signal at the bottom of the MTS. Spectra were taken using a 785 nm laser excitation, 1 s integration time and 100% laser power at 20 mW. All measurements were taken on *n* = 3 MTS.

Since the two sets of nanotags had very similar distribution within the MTS, and no additional information was found by changing the incubation time and concentration, more in depth SERS optical mapping was carried out. The previous XZ SERS maps were captured using a low *z* resolution (50 μm); therefore, a series of higher *z* resolution (1 μm) 3D maps were obtained throughout the entire depth of the MTS. A grid of 100 μm using *x* = 10 μm, and *y* = 10 μm in the centre of the spheroid was created. For greater clarity, these SERS maps were plotted on a logarithmic scale (Fig. 2). When plotted linearly, the signal arising from the bottom of the spheroid was seldom observed within the images, due to the high intensity of the SERS signal at the top of the MTS. Therefore, this did not enhance the absence of signal within the core as there was no clear difference between the core and the bottom. However, by plotting it on the logarithmic scale, it becomes possible to show the presence of signal at the bottom of the spheroid and correlate it to the *z* profile observed (Fig. 2B) and the true location of the nanotags more clearly. The resulting SERS images presented in Fig. 2A gave a clearer representation of the location of the nanotags within the MTS and fully supported the previous experimental findings that the nanotags were distributed throughout the periphery of the MTS. The same trend was observed for the cAb nanotags (data is not shown). Plotting the *z* profile (Fig. 2B) further strengthened this conclusion that the nanotags were localising within the outer layer of the MTS and did not appear to be entering the core. Since there is a high intensity at the top of the MTS, reduction in signal in the middle, then an eventual increase again, corresponding to the bottom of the MTS, these high-resolution depth scans further support that the nanotags were unable to penetrate to the core of the MTS.

After this higher resolution experiment, we were confident that the nanotags were not penetrating the core of the MTS because the higher resolution map would have detected them much more accurately than the lower resolution maps in Fig. 1. In general, even though NPs with diameters approaching 100 nm are able to enter cells, the size of NPs which are able to penetrate the core of spheroids are reported to be <30 nm.^15,17^ Since both sets of nanotags were 77 nm and 76 nm in diameter, it was concluded that one reason they did not further penetrate the spheroid core was due to their size. The nanotags are initially up taken by the cells in the proliferating layer. However, due to their larger size, they become “stuck” within the cells that they initially enter, and do not exit the cell to travel deeper into the spheroid. Therefore, this is an important aspect to consider when investigating the uptake of NPs into MTS post formation rather than incubating a 2D monolayer of cells with NPs. It is proposed that due to the monolayer of single cells being available for NP uptake, that there would be a greater distribution of NPs within each of the cells of the MTS if it was formed after NP incubation. Whereas in this case, it was found the NPs were not able to travel past the outer proliferating layer of the MTS.

Furthermore, the cells in an MTS become hypoxic and necrotic as they approach the core, due to being further away from the available oxygen and nutrients in the surrounding medium. Under such conditions, the production of adenosine triphosphate (ATP) is diminished, and since the majority of NP uptake into cells is through active transport,^10^ which requires ATP, the uptake would be significantly reduced in hypoxic or necrotic cells. Since the two sets of nanotags were showing similar uptake results, regardless of the conjugation of an antibody specific to tenascin-C overexpressed by the U87-MG glioblastoma cell line or a control antibody, it was necessary to further examine the nature of the cells within the MTS.

**Fig. 3:**
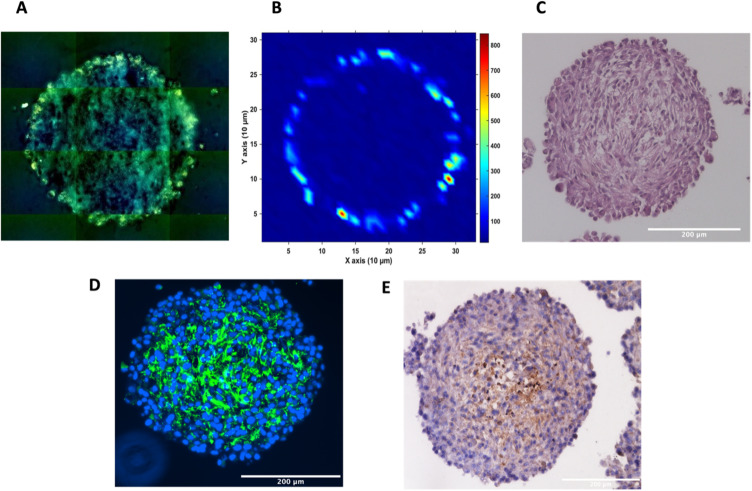
Sectioned U87-MG GBM MTS. (A) White light montage image taken on a Renishaw InVia Raman microscope of the MTS section to illustrate the nanotags around the proliferating layer of the MTS incubated with 32 pM Ab nanotags and (B) a corresponding SERS intensity map using the 956 cm^−1^ PPY peak, (C) haematoxylin and eosin (H&E) staining, (D) immunofluorescence, where the presence of the green indicates tenascin-C (E) immunohistochemistry, where the presence of the brown colour indicates tenascin-C. (A) and (B) are of the same MTS section. (C–E) are sections of different MTS, however prepared at the same time and under the same conditions. SERS intensity map was taken using a 785 nm laser excitation, 1 second integration and a laser power of 10 mW. All measurements were taken on *n* = 3 MTS.

To investigate the distribution of the nanotags further and obtain a clearer indication of any uptake throughout the depth of the MTS, and the morphology of the cells within the MTS, a series of fixed and paraffin embedded MTS were sectioned to produce 5 μm-thick sections. Cross sectioning the MTS allows for a better observation of the nanotags, minimises interference from the MTS during SERS mapping, and allows for immunostaining and visualisation of the cells (Fig. 3). Details regarding the cross sectioning and immunostaining are provided in the ESI.† As observed in Fig. 3A, white light images of the MTS sections under the microscope showed gold-like colouring in the outer portions of the MTS suggesting the presence of the nanotags throughout the outer layer of cells as indicated by the scattered light. The corresponding SERS map (Fig. 3B) for PPY showed that the SERS signal, and thus the nanotags, were distributed only in the first 20–40 μm of the spheroid. The results of these 2D cross-sections clearly agreed with the SERS maps obtained by optical sectioning shown in Fig. 1 and 2 that the nanotags are not able to penetrate beyond the outer proliferating layer of the MTS.

To gain further insight into the MTS themselves, a series of immunohistological stains were used. Haematoxylin and eosin (H&E) staining was carried out to identify the nucleus (haematoxylin, deep purple) and the cytoplasm (eosin, light purple/pink) of the sectioned MTS (Fig. 3C). This staining indicated the compactness of the cells within the spheroid. The H&E images also agree with other studies demonstrating that the outer compressed layer represents proliferating cells.^37^ Immunofluorescence (IF) and immunohistochemistry (IHC) were carried out to demonstrate the presence of tenascin-C within the U87-MG MTS sections. A primary rabbit monoclonal antibody to tenascin-C was added to the sections and for IF, a goat anti-rabbit fluorophore (Alexa488) secondary antibody was then added giving rise to a green colour indicating the presence of tenascin-C, in Fig. 3D. A secondary, horseradish peroxidase (HRP) conjugated antibody was added for IHC and the presence of the brown colour indicates the presence of tenascin-C (Fig. 3E). This staining indicated that the tenascin-C was present within the stroma of the cells; a finding that is also supported by the literature.^38^ These images provided evidence that the tenascin-C was expressed in greater quantities in the central region of the spheroid, shown by higher quantities of the green and brown colours (Fig. 3D and E).

**Fig. 4:**
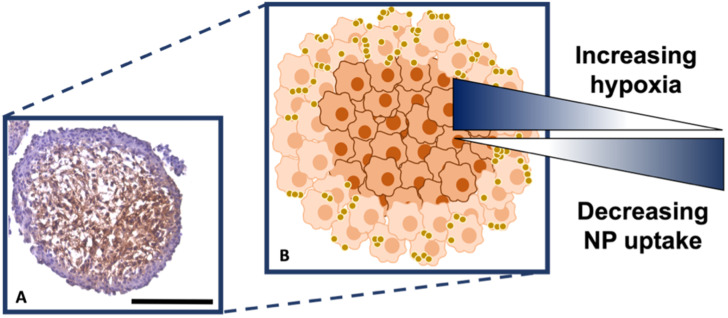
(A) Sectioned MTS showing hypoxic staining using pimonidazole, where the presence of the brown colour indicates hypoxia. (B) Schematic correlating hypoxia and NP uptake within the MTS based on (A). The outer, actively proliferating layer of cells of the MTS are coloured light orange, the quiescent and hypoxic cells are dark orange. The gold spheres illustrate the nanotags. Scale bar is 200 μm.

It is known that hypoxia upregulates tenascin-C expression,^39^ therefore, pimonidazole hydrochloride was used to determine the presence of hypoxic cells within the cross-section. Fig. 4A indicates the MTS section, with the central portion of the MTS stained brown, indicating the presence of hypoxia, whereas the outer portions of the MTS remain purple from counterstained haematoxylin. This proliferating layer was found to be approximately 40 μm in size and therefore this fully correlates to the NP uptake observed in the white light and SERS intensity images of the MTS in Fig. 3A and B, where the NP uptake was approximately 20–40 μm and therefore not being up taken beyond the proliferating layer of cells. This further supports the data that the NPs remain within the proliferating layer of the spheroids and do not penetrate the quiescent layer or hypoxic core, despite the tenascin-C expression clearly being higher within the regions closer to the core of the MTS. The reduction in AuNP uptake was also hypothesised to be partly due to an inefficient anaerobic glycolytic metabolism under hypoxic conditions, resulting in a lower level of ATP production and therefore NP uptake.^40^ It was therefore hypothesised that a lack of ATP resulted in reduced active targeting, and therefore NP uptake, because the cells were not actively proliferating due to being either quiescent or hypoxic. This is a highly important observation to make as the 2D monolayer data (Fig. S3†) suggested there were no issues with cellular uptake. However, when translating to the MTS, and therefore exposing complications such as the compactness of cells and hypoxia, it was shown that cellular uptake is clearly hindered. As such, this emphasises the importance of investigating cellular uptake *in vitro* for both 2D cells and 3D models prior to *in vivo* research.

Therefore, despite the addition of the tenascin-C antibody onto the surface of the AuNP-SHINs, the nanotag size, MTS compactness and the variations in cell proliferation and oxygenation state have clearly compromised the ability of the nanotags to penetrate the MTS. We have shown that the uptake of nanotags, both with and without the targeting antibody, is similar and propose the following explanation. Firstly, the proliferating cells appeared to be more compact than the cells within the core of the MTS (Fig. 3C), and therefore this compactness could restrict the nanotags from being able to pass through the interstitial spaces between cells. Again, given the size of the nanotags (∼80 nm), it is possible that they are unable to travel past the proliferating layer of the spheroid^13^ due to the cell packing causing them to become localised in the cells forming the outer edge of the spheroid. Even though the nanotags are ∼80 nm and nearing the upper limit of the size range for being up taken by cells,^11,41^ the Raman and SERS maps clearly indicated that the nanotags were up taken by the outer cells as observed in the SERS intensity maps of the nanotags correlating with the Raman map for the cellular stretches (Fig. 1C). Therefore, our results indicated that the nanotags are being up taken into the cells of the spheroid. Additionally, since the size of the nanotags seem to be hindering their penetration into the deeper layers of the spheroids, where the immunohistochemistry indicated that the tenascin-C expression levels are higher, this could be a reason there does not seem to be different uptake between the Ab and the cAb nanotags. Finally, another possible issue is the uptake of nanotags. If the nanotags can reach the core, it would be expected that a stronger SERS signal would be detected due to the increased levels of tenascin-C expression. However, it is known that the presence of hypoxia in tumours can reduce cellular uptake due to dense stroma, abnormal angiogenesis and interstitial pressure, making it difficult for NPs to reach the hypoxic cells.^42^ Additionally, the binding of the NPs to hypoxic cells is challenging, further reducing the probability of cellular uptake.^42^ Therefore, regardless of the tenascin-C expression levels, very few nanotags would be able to enter the core. Nevertheless, we propose the following model of the spheroids upon incubation with the nanotags (Fig. 4B). This model applies for formed MTS that are subsequently incubated with nanotags, as opposed to single cells being incubated with nanotags that are then used to prepare the MTS. It considers both the distribution of the nanotags and the hypoxic state of the cells within the MTS. This schematic in Fig. 4B as a general model was observed several times^15,17^ when incubating NPs into 3D MTS where the NPs are residing in the outer layer of the spheroids and a general consensus was that penetration is size dependent. Since this represents the uptake of the NPs on an *in vitro* 3D model, due to the similarities mentioned between the MTS and an *in vivo* tumour, it is thought that a similar effect would occur *in vivo*, and targeting could be limited to the outer proliferating cells. From the literature, it seems that AuNP uptake into 3D MTS is an important area still not fully understood that depends on NP size, shape, surface chemistry and differences between cell lines of varying origin.

## Conclusions

We have shown the use of SERS imaging to locate and demonstrate successful uptake of nanotags within formed U87-MG glioblastoma MTS. Assessing NP uptake in MTS is more representative of the scenario *in vivo* compared to assessing uptake in a monolayer of cells or creating MTS from cells already impregnated with NPs. The data demonstrated that rather than having NPs distributed throughout the spheroid, the nanotags were localised to the outer layers of the MTS. We attribute this distribution to a combination of nanotag size relative to the tight packing of the cells within the spheroid, and the hypoxic nature of the spheroid core, leading to a reduction in the production of ATP and NP binding. Therefore, regardless of the targeting antibody, the nanotags are accumulating within the proliferating layer of the MTS and further studies are required to investigate the effects of different targeting ligands and different sizes and shapes of NPs. Overall, this work provides new insights into how AuNPs distribute themselves within oxygenated and hypoxic cells, such as those within 3D cancer MTS, and how SERS is a useful non-destructive tool that provides information on NP uptake and distribution. MTS are a representative bridge between 2D cellular culture and *in vivo* models; however, they do not fully reflect the *in vivo* tumour. Therefore, due to the complexity observed of the uptake of AuNPs in 3D models, let alone in *in vivo* models, more research is required to understand nanotag uptake and targeting in already formed *in vitro* 3D models before carrying out *in vivo* experiments.

## Data availability

The research data associated with this paper is available at the following link: 10.15129/1e94403e-31a4-432d-9bdd-7a1b8ff75cc8.

## Conflicts of interest

There are no conflicts to declare.

## Supplementary Material

AN-148-D3AN00751K-s001

AN-148-D3AN00751K-s002

AN-148-D3AN00751K-s003

AN-148-D3AN00751K-s004

AN-148-D3AN00751K-s005

AN-148-D3AN00751K-s006

AN-148-D3AN00751K-s007

AN-148-D3AN00751K-s008

AN-148-D3AN00751K-s009

## References

[cit1] Cancer Research UK, https://www.cancerresearchuk.org/health-professional/cancer-statistics-for-the-uk

[cit2] Cancer Research UK, https://www.cancerresearchuk.org/health-professional/cancer-statistics/statistics-by-cancer-type/brain-other-cns-and-intracranial-tumours

[cit3] Trédan O., Galmarini C. M., Patel K., Tannock I. F. (2007). JNCI, J. Natl. Cancer Inst..

[cit4] Bowers H. J., Fannin E. E., Thomas A., Weis J. A. (2020). Sci. Rep..

[cit5] Kunz-Schughart L. A., Kreutz M., Knuechel R. (1998). Int. J. Exp. Pathol..

[cit6] McMillan K. S., McCluskey A. G., Sorensen A., Boyd M., Zagnoni M. (2016). Analyst.

[cit7] Däster S., Amatruda N., Calabrese D., Ivanek R., Turrini E., Droeser R. A., Zajac P., Fimognari C., Spagnoli G. C., Iezzi G., Mele V., Muraro M. G. (2017). Oncotarget.

[cit8] Jamieson L. E., Harrison D. J., Campbell C. J. (2015). Analyst.

[cit9] Popp M. K., Oubou I., Shepherd C., Nager Z., Anderson C., Pagliaro L. (2014). J. Nanomater..

[cit10] Kapara A., Brunton V., Graham D., Faulds K. (2020). Chem. Sci..

[cit11] Chithrani B. D., Ghazani A. A., Chan W. C. W. (2006). Nano Lett..

[cit12] Xia Q., Huang J., Feng Q., Chen X., Liu X., Li X., Zhang T., Xiao S., Li H., Zhong Z., Xiao K. (2019). Int. J. Nanomed..

[cit13] Huo S., Ma H., Huang K., Liu J., Wei T., Jin S., Zhang J., He S., Liang X.-J. (2013). Cancer Res..

[cit14] Ricketts K. P. M., Cheema U., Nyga A., Castoldi A., Guazzoni C., Magdeldin T., Emberton M., Gibson A. P., Royle G. J., Loizidou M. (2014). Small.

[cit15] Bromma K., Alhussan A., Perez M. M., Howard P., Beckham W., Chithrani D. B. (2021). Cancers.

[cit16] Rane T. D., Armani A. M. (2016). PLoS One.

[cit17] Ahmed-Cox A., Pandzic E., Johnston S. T., Heu C., McGhee J., Mansfeld F. M., Crampin E. J., Davis T. P., Whan R. M., Kavallaris M. (2022). J. Controlled Release.

[cit18] Tucić M., Stamenković V., Andjus P. (2021). Front. Cell Dev. Biol..

[cit19] Fu Z., Zhu G., Luo C., Chen Z., Dou Z., Chen Y., Zhong C., Su S., Liu F. (2022). Front. Oncol..

[cit20] Marzeda A. M., Midwood K. S. (2018). J. Histochem. Cytochem..

[cit21] Midwood K. S., Chiquet M., Tucker R. P., Orend G. (2016). J. Cell Sci..

[cit22] Orend G., Chiquet-Ehrismann R. (2006). Cancer Lett..

[cit23] Zhang Q., Xu B., Hu F., Chen X., Liu X., Zhang Q., Zuo Y. (2021). J. Mol. Neurosci..

[cit24] Lingasamy P., Tobi A., Kurm K., Kopanchuk S., Sudakov A., Salumäe M., Rätsep T., Asser T., Bjerkvig R., Teesalu T. (2020). Sci. Rep..

[cit25] Kapara A., Findlay Paterson K. A., Brunton V. G., Graham D., Zagnoni M., Faulds K. (2021). Anal. Chem..

[cit26] Fleischmann M., Hendra P. J., McQuillan A. J. (1974). Chem. Phys. Lett..

[cit27] Han X. X., Rodriguez R. S., Haynes C. L., Ozaki Y., Zhao B. (2022). Nat. Rev. Methods Primers.

[cit28] Kearns H., Goodacre R., Jamieson L. E., Graham D., Faulds K. (2017). Anal. Chem..

[cit29] Laing S., Gracie K., Faulds K. (2016). Chem. Soc. Rev..

[cit30] Altunbek M., Çetin D., Suludere Z., Çulha M. (2019). Talanta.

[cit31] Nicolson F., Jamieson L. E., Mabbott S., Plakas K., Shand N. C., Detty M. R., Graham D., Faulds K. (2018). Analyst.

[cit32] Li J. F., Tian X. D., Li S. B., Anema J. R., Yang Z. L., Ding Y., Wu Y. F., Zeng Y. M., Chen Q. Z., Ren B., Wang Z. L., Tian Z. Q. (2013). Nat. Protoc..

[cit33] Krajczewski J., Kudelski A. (2019). Front. Chem..

[cit34] Turkevich J., Stevenson P. C., Hillier J. (1951). Discuss. Faraday Soc..

[cit35] Tipping W. J., Wilson L. T., An C., Leventi A. A., Wark A. W., Wetherill C., Tomkinson N. C. O., Faulds K., Graham D. (2022). Chem. Sci..

[cit36] Rossi M., Blasi P. (2022). Front. Med. Technol..

[cit37] Kunz-Schughart L. A., Kreutz M., Knuechel R. (1998). Int. J. Exp. Pathol..

[cit38] Xia S., Lal B., Tung B., Wang S., Goodwin C. R., Laterra J. (2016). Neuro-Oncology.

[cit39] Midwood K. S., Orend G. (2009). J. Cell Commun. Signaling.

[cit40] Jain S., Coulter J. A., Butterworth K. T., Hounsell A. R., McMahon S. J., Hyland W. B., Muir M. F., Dickson G. R., Prise K. M., Currell F. J., Hirst D. G., O'Sullivan J. M. (2014). Radiother. Oncol..

[cit41] Hoshyar N., Gray S., Han H., Bao G. (2016). Nanomedicine.

[cit42] Li X., Wu Y., Zhang R., Bai W., Ye T., Wang S. (2021). Front. Mol. Biosci..

