# Supplementary material for: Evaluating nanoparticle localisation in glioblastoma multicellular tumour spheroids by surface enhanced Raman scattering
Source: Analyst. 2023 Jun 16;148(14):3247–56. doi: 10.1039/d3an00751k (PMC10332387; doi:10.1039/d3an00751k)
Supplement: AN-148-D3AN00751K-s002 [file AN-148-D3AN00751K-s002.pdf]

Electronic Supplementary Material (ESI) for Analyst.
This journal is © The Royal Society of Chemistry 2023

Evaluating Nanoparticle Localisation in Glioblastoma Multicellular Tumour Spheroids by Surface-Enhanced Raman Spectroscopy

Samantha M. McCabe[a,b], Gregory Q. Wallace[a], Sian Sloan-Dennison[a], William J. Tipping[a], Neil C. Shand[b], Duncan Graham[a], Marie Boyd[c], Karen Faulds[a*]

[a.] *Department of Pure and Applied Chemistry, Technology and Innovation Centre, University of Strathclyde, 99 George Street, Glasgow, G1 1RD, UK.*

[b.] *The Defence Science and Technology Laboratory (Dstl), Porton Down, Salisbury, SP4 0JQ, UK.*

[c.] *Strathclyde Institute of Pharmacy and Biomedical Sciences, University of Strathclyde, 161 Cathedral Street, Glasgow, G4 0RE, UK.*

SUPPLEMENTARY INFORMATION

Materials and Methods

Synthesis of SERS nanotags

50 nm gold nanoparticle (AuNP) seeds were synthesised using a slightly modified Turkevich citrate reducing method to produce gold spherical NPs.[30] Sodium tetrachloroaurate(III) dihydrate, (60.5 mg) was dissolved in 500 mL of doubly distilled water (ddH_2O) and heated until boiling under continuous stirring. Sodium citrate tribasic dihydrate (57.5 mg) was dissolved in 7.5 mL dH_2O and added to the mixture. This was boiled with continuous stirring for 15 minutes and the resulting solution was left to cool with continuous stirring.

Shell isolated AuNP (AuNP-SHIN) with a diameter of 58 nm were synthesised using the 50 nm seeds (100 mL, 300 nM) and (4-(1H-pyrazol-4-yl) pyridine (PPY) was added as the Raman reporter. The silica shell was formed by adding (3-aminopropyl)trimethoxysilane (APTMS) (150 µL, 1 mM) quickly followed by sodium silicate (1.5 mL, 32 µM), the solution was placed in a 90 ºC oil bath for 30 minutes with stirring. The mixture was then left to cool with stirring overnight. The resulting NPs were concentrated by centrifugation (6000 RPM, 20 min) and resuspended in 30 mL of dH_2O.

Antibody conjugation

For antibody conjugation, 50 µL of borate buffer (pH = 9) was added to 500 µL of PPY-AuNP-SHINs. 5 µL of a monoclonal to tenascin-C antibody (0.5 mg/mL) (ab109830) was added to the mixture and shaken for 2 hours on a shaking plate. Bovine serum albumin (BSA) (100 mg/mL, 40 µL) was added as a protection layer to the surface of the NPs with shaking for 30 minutes. The resulting NP conjugates were concentrated by centrifugation (4000 RPM, 20 min) and resuspended in 450 µL of dH_2O. This procedure was repeated for the goat anti-rabbit IgG (0.5 mg/mL) (ab6702) non-specific antibody control. A lateral flow immunoassay was carried out to ensure that the targeting antibody (rabbit) was present on the surface of the nanotags. The nitrocellulose lateral flow strips were pre-conjugated with a goat anti-rabbit IgG (ab6702) antibody. If a line appeared, then the targeting antibody was successfully conjugated to the nanotags. A control strip was also run alongside, without addition of the antibody.

Characterisation

Extinction spectroscopy: Extinction spectroscopy was carried out using an Agilent, Cary 60 UV-Visible spectrophotometer. A 1 cm pathlength poly(methylmethacrylate) (PMMA) plastic cuvette was used, and a baseline was obtained prior to running the samples using dH_2O.

Size and Zeta Potential measurements: Dynamic light scattering (DLS) and zeta measurements were obtained using a Malvern Zetasizer, Nano ZS system (Malvern, UK). Before sample analysis, a 40 nm

polystyrene latex bead standard was run to calibrate the instrument. To measure the size, the NPs were placed into a 1 cm PMMA cuvette. The samples were measured in triplicate and the average mean and standard deviation was recorded. The zeta potential of the samples was measured using a dip cell placed into the 1 cm PMMA cuvette.

SERS measurements: SERS measurements to confirm the presence of the Raman reporter and successful synthesis of the AuNP-SHINs were taken on a handheld CBex Snowy Range instrument with a 785 nm laser excitation, laser power 75 mW, integration time of 0.2 s.

Nanoparticle tracking analysis (NTA): A Nanosite 300 instrument with NTA software was used to assess the concentration of the AuNP-SHIN nanotags. The measurements were performed at room temperature (RT) and 60 second movies were created for each sample, which was repeated 5 times. The camera level was set to 6 and the NPs were focussed. The sample addition was by a 1 mL syringe set in a syringe pump, with a flow rate of 100. The number of nanotags over 20 frames of a 60 second video were counted and the average number of particles per frame was calculated, giving to the number of nanoparticles per mL.

Staining

Haematoxylin and Eosin (H&E): The spheroid sections were added to histoclear to remove the paraffin wax then rehydrated in decreasing concentrations of ethanol (100%, 90% and 70%) for 2 minutes each. They were then fixed in ice cold acetone:ethanol (30:70) for 15 minutes. Next, they were placed into tris-buffered saline (TBS) for 10 minutes for washing. They were then placed into haematoxylin for 10 minutes, washed in running tap water, dipped in Scott's tap water then placed into eosin stain for 2 dips and washed in running water until the desired colour was observed. The sections were then rehydrated again in increasing concentrations of ethanol (70%, 90% and 100%) before being mounted to a glass coverslip using DPX mounting medium. The sections were imaged using an EVOS FL Auto system (Life technologies, UK).

Immunohistochemsitry: The spheroid sections were added to histoclear to remove the paraffin wax then rehydrated in decreasing concentrations of ethanol (100%, 90% and 70%) for 2 minutes each. For antigen retrieval, the sections were placed into retrieval buffer (10 mM sodium citrate, 0.05% Tween20 at pH 6) and placed into a plastic pressure cooker and heated in the microwave for 10 minutes at the highest power then left to cool for 20 minutes until reaching RT. They were then washed in tris-buffered saline with Tween20 (1%) (TBS-T) on a rocker for 10 minutes. The slides were then added to 5% bovine serum albumin (BSA) in TBS-T for 30 minutes for blocking and washed again in TBS-T for 10 minutes on a rocker. Rabbit monoclonal antibody to tenascin-C (primary antibody) was added (diluted 1:200 in TBS-T) and left in a humidified chamber at 4 ºC overnight. The sections were washed with TBS-T on a rocker for 10 minutes and then a goat anti-rabbit HRP conjugated secondary antibody

(ab6721) was added for 1 hour in a humidified chamber at RT. Sections were developed using DAB substrate kit (ab64238) for 10 minutes at RT, sections were washed to stop the reaction with the DAB and counterstained in haematoxylin before being mounted to a glass coverslip using DPX mounting medium. The sections were imaged using an EVOS FL Auto system (Life technologies, UK).

Immunofluorescence: The spheroid sections were added to histoclear to remove the paraffin wax then rehydrated in decreasing concentrations of ethanol (100%, 90% and 70%) for 2 minutes each. For antigen retrieval, the sections were placed into retrieval buffer (10 mM sodium citrate, 0.05% Tween20 at pH 6) and placed into a plastic pressure cooker and heated in the microwave for 10 minutes at the highest power then left to cool for 20 minutes until reaching RT. They were washed in TBS-T on a rocker for 10 minutes. They were then added to 5% BSA in TBS-T for 30 minutes for blocking and washed again in TBS-T for 10 minutes on a rocker. Rabbit monoclonal antibody to tenascin-C (primary antibody) was added (diluted 1:200 in TBS-T) and left in a humidified chamber at 4 ºC overnight. The sections were washed with TBS-T on a rocker for 10 minutes and then a goat anti-rabbit IgG (Alexa 488) (ab150077) secondary antibody was added for 1 hour in a humidified chamber. After, the sections were washed in TBS-T on a rocker for 15 minutes. For nuclei stain, DAPI- mounting medium was added to the slides. Coverslips were added on top and sealed. The sections were imaged using an EVOS FL Auto system (Life technologies, UK) for both DAPI (405 nm) and ALEXA488 (488 nm).

Detection of Hypoxia: Hypoxia in the spheroids was detected using the Hypoxyprobe Plus Kit containing FITC conjugated to anti-pimonidazole mouse IgG, monoclonal antibody (FITC-MAb1) and a rabbit anti-FITC conjugated with HRP as a secondary agent. The spheroids were grown as normal and a 100 μM pimonidazole hydrochloride solution was then added to them to allow for incubation for 2 hours on a roller to allow the pimonidazole to form adducts with thiol containing proteins in the spheroids that have an oxygen concentration of less than 14 μM which is equivalent to a partial pressure of pO_2 = 10 mm Hg at 37 ºC. The spheroids were then sectioned using a microtome as described. The sections were added to histoclear to remove the paraffin wax then rehydrated in decreasing concentrations of ethanol (100%, 90% and 70%) for 2 minutes each. They were washed in tris-buffered saline with Tween20 (0.1%) TBS-T for 10 minutes. They were added to 3% H_2O_2 in dH_2O for 5 minutes and washed in TBS-T. For antigen retrieval, the sections were placed into retrieval buffer (10 mM sodium citrate, 0.05% Tween20 at pH 6) and placed into a pressure cooker. They were heated in the pressure cooker in the microwave for 10 minutes at the highest power then left to cool for 20 minutes until reaching RT. The sections were washed with TBS-T and then added to 5% BSA in TBS-T for 10 minutes for blocking. The primary antibody (FITC-MAb1) was added (diluted 1:100 in TBS-T) and left in a humidified chamber for 1 hour. The sections were washed with TBS-T for 10 minutes and then rabbit anti-FITC conjugated with HRP (diluted 1:100 in TBS-T) was added for 30 minutes in a humidified chamber. The sections were then washed in TBS-T for 2 minutes. Sections were developed

using DAB substrate kit (ab64238) for 10 minutes at RT, washed with H_2O, counter stained with haematoxylin and coverslips were added on top and sealed with mounting media. The sections were imaged using an EVOS FL Auto system (Life technologies, UK).

Data processing

Processing of SERS Spectra: After having been acquired, all spectra were processed using Python 3. For each SERS spectrum, a 3rd order polynomial baseline was determined and subtracted from the original spectrum, yielding a baseline corrected spectrum. No smoothing was applied to the spectra. The resulting SERS maps were generated by finding the maximum peak intensity within a narrow range surrounding the peak of interest (~ ±20 cm^{-1}) for each pixel in the map. Using a narrow range as opposed to the same Raman shift accounts for any slight fluctuation in the spectral position of the maximum intensity. The map was then reconstructed using MatLab (R2020b, Mathworks, USA) software.

Direct Classical Least Squares (DCLS): For the DCLS data processing, WiRE 4.4 (Renishaw, UK) software was used to remove any cosmic rays and conduct a baseline correction. For cosmic ray removal, WiRE applies a simple zap function to replace any sharp, cosmic rays. For baseline corrections, WiRE conducts an "intelligent" polynomial fitting software. WiRE creates false colour images of the SERS map using a direct classical least squares (DCLS) method. This method is used when there is a reference spectrum to compare the data collected to. If there is a good spectral match between the two spectra, then the false colour image is created. The presence of the false colour indicates the presence of the nanotags within the cells. The spectra obtained were compared to a reference PPY spectra of the nanotags using intensity at point of the exact cell. By observing the spectra from different areas of the cell where the spectrometer provided a false colour image for the nanotags, it was possible to compare this to the reference spectra to assess nanotag accumulation within the cell.

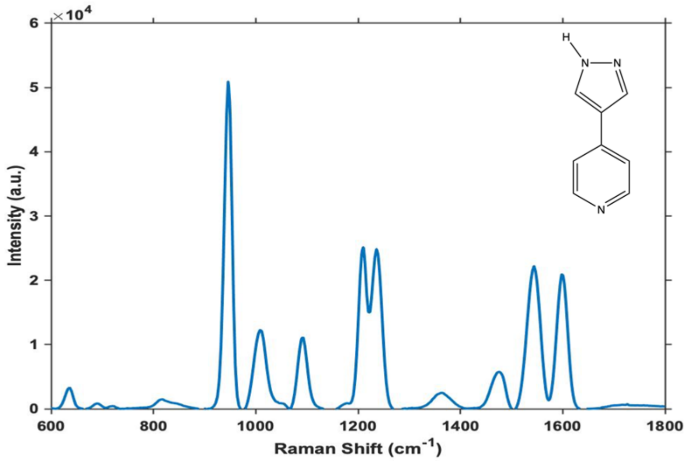

Fig. S1: SERS spectrum of 4-(1H-pyrazol-4-yl) pyridine (PPY) taken on a CBex Snowy Range handheld Raman spectrometer. A 785 nm laser excitation was used with a laser power of 75 mW and an integration time of 0.2 seconds. The structure of PPY is included.

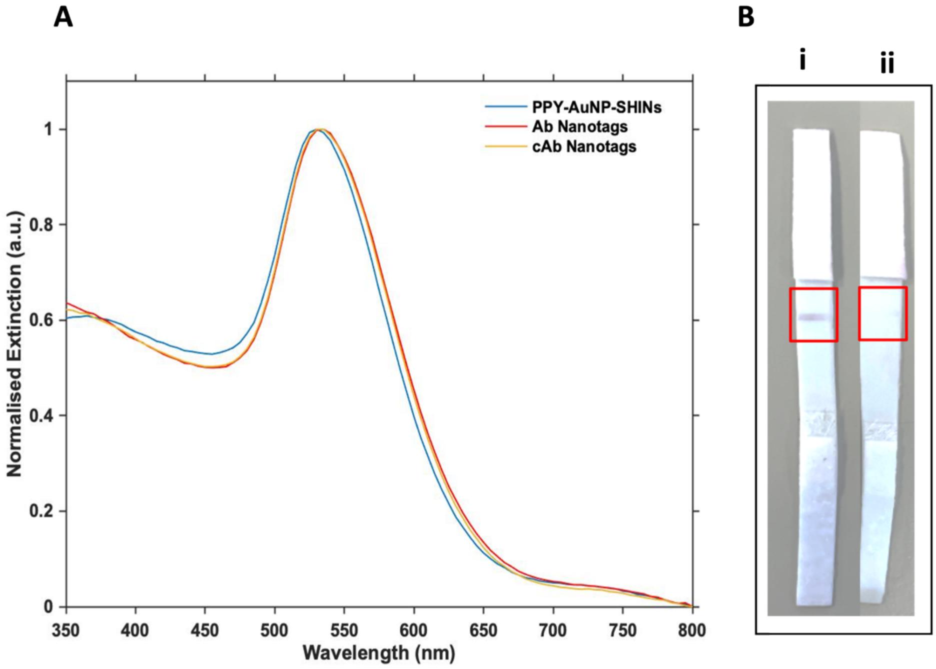

Fig. S2: (A) Normalised extinction spectra of PPY-AuNP-SHINs (blue, diluted 1 in 4), Ab nanotags (red, undiluted), and cAb nanotags (yellow, undiluted). (B) Lateral flow immunoassay where i) shows the presence of a line and therefore binding of the Ab nanotags, ii) shows the absence of a line and therefore the lack of binding of the nanotags with no Ab attached identified by the red marker.

Table S1: Dynamic light scattering (DLS) and zeta potential measurements for PPY-AuNP-SHINs, Ab nanotags and cAb nanotags used

	PPY-AuNP-SHINs	Ab Nanotags	cAbNanotags
Size (nm)	58 ± 2	77 ± 3	76 ± 2

**Zeta Potential (mV)**	**-44 ± 3**	**-24 ± 1**	**-23 ± 0.6**

Given the large size of the nanotags (~80 nm), it was necessary to verify their uptake into the U87-MG cells that were to be used to make the MTS. Single cell analysis was carried out on both the Ab and cAb nanotags (Fig. S3). Raman microscopy measurements were conducted using 532 and 785 nm laser excitation wavelengths to understand the uptake and location of the NPs in the cells and MTS. The 532 nm excitation wavelength was used to identify cellular stretches by Raman and 785 nm was used to detect the SERS spectrum from the nanotags. Both sets of nanotags were added to a 2D monolayer of U87-MG cells at 32 pM and cultured in an incubator set to 37 ºC with 5% CO_2 for 4 hours. The nanotags were removed and the cells were washed with phosphate buffered saline (PBS) and fixed with 4% paraformaldehyde (PFA) for 15 minutes at room temperature (RT) then stored in PBS for mapping. SERS XY maps were created by mapping three different planes (z = +5 µm, z = 0 µm and z = -5 µm) of each single cell (n=3), where x = 2.5 µm and y = 2.5 µm. This was carried out to illustrate that there was nanotag uptake present within various planes of the cell and therefore successfully presented throughout. The presence of the purple colour from false colour DCLS mapping in each z axes indicates the presence of the Ab and cAb nanotags throughout the cell volume. Since this false colour is present in each plane at different areas and levels, this suggested that there are different nanotags present within each layer. Additionally, creating an XY Raman map, where x = 2.5 µm, y = 2.5 µm and z = 0, allows for the correlation of the cellular stretch to match that of the PPY nanotags and confirm that they are within the cells.

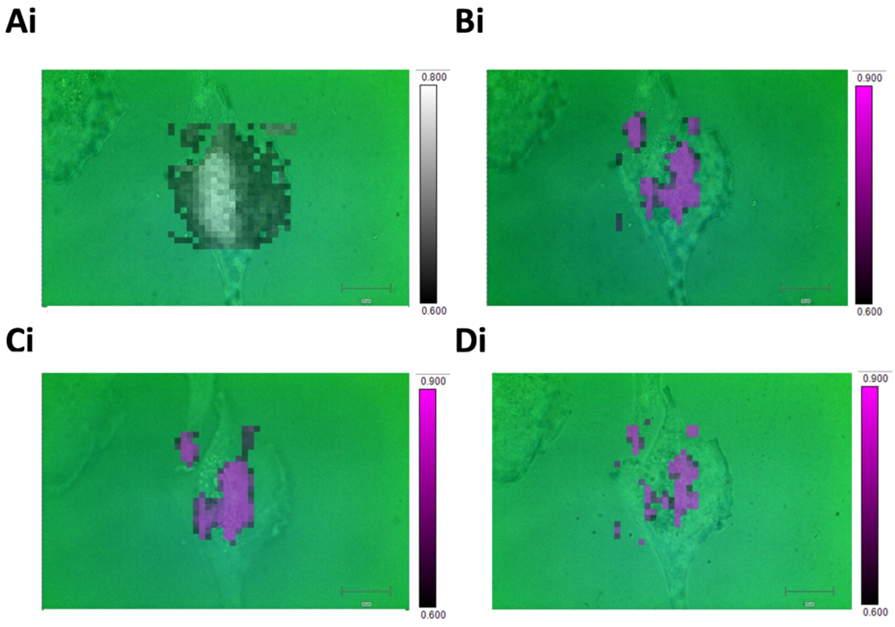

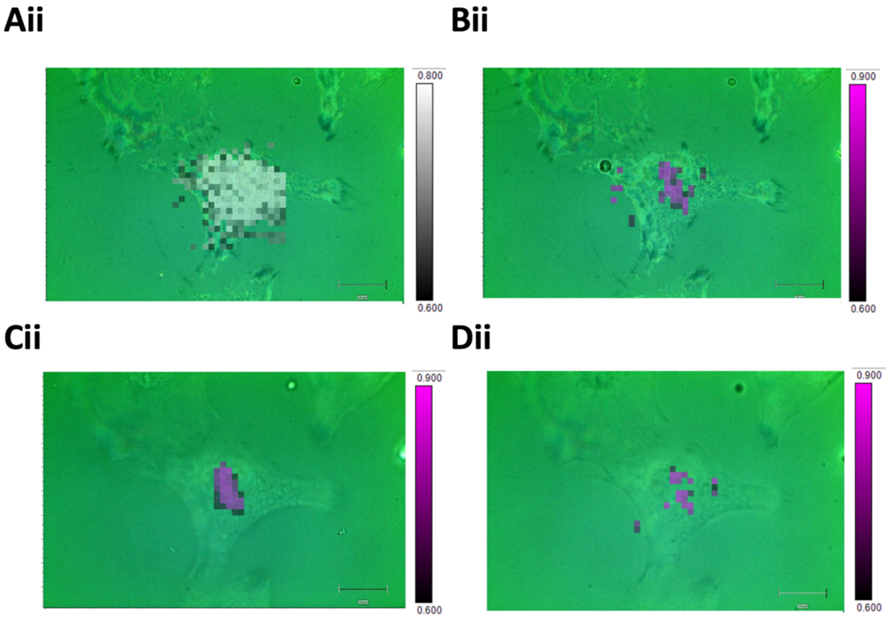

Fig. S3 2D direct classical least squares (DCLS) single U87-MG cell uptake study on carried out on n=3 cells (i) Ab nanotags and (ii) cAb nanotags. (A) Tracking of the 2930 cm^{-1} peak of the CH_3 protein cellular stretch, using a 532 nm laser excitation. (B-D) Tracking of the main PPY peak at 956 cm^{-1}, using a 785 nm laser excitation through three different planes of the cell. All spectra were taken using a 2.5 µm step size, 1 s integration time and 100% laser power (785 nm, 20 mW and 532 nm, 30 mW).

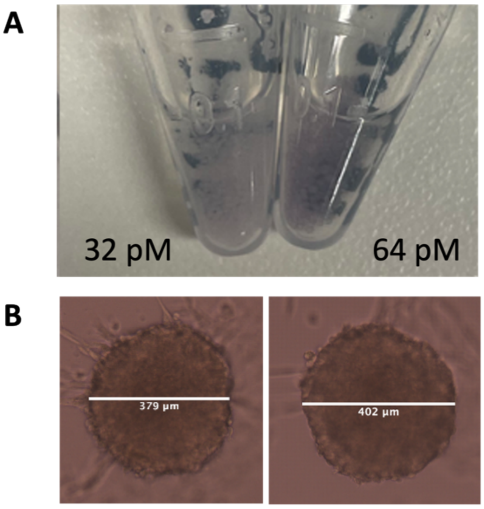

Fig. S4: (A) Example of the physical colour change of the MTS after incubation with the Ab nanotags at 32 pM and 64 pM. Clearly, an increase in concentration results in a deeper purple colour. (B) Examples of MTS with their size indicated.

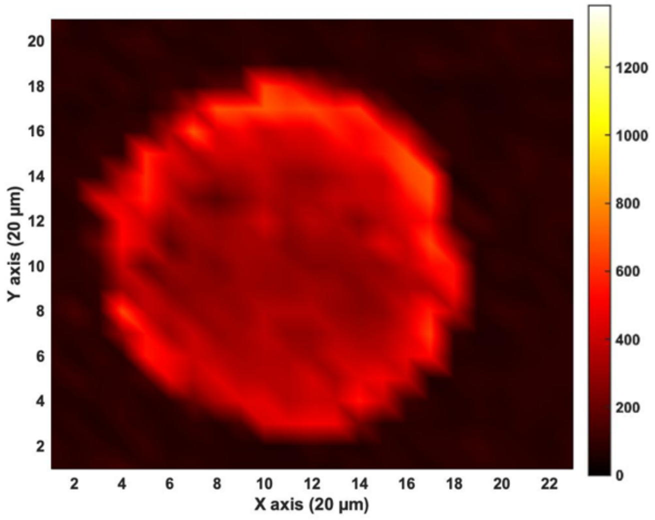

Fig S5: 2D XY Raman intensity map at 2930 cm^{-1} showing the CH_3 cellular protein peak carried out on n = 3, obtained using 532 nm laser excitation taken at the middle of the spheroid used in Fig. 1. Spectra were taken using a 20 µm step size, 1 s integration time and 100% laser power (30 mW), with a 532 nm laser excitation.

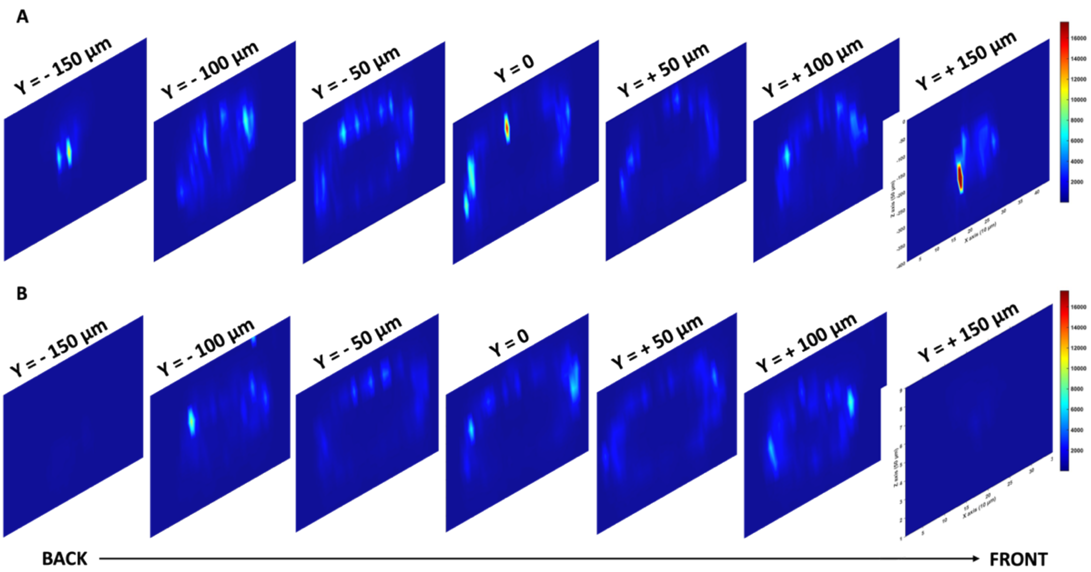

Fig. S6 2D XZ SERS intensity maps at 956 cm^{-1} corresponding to the main intensity peak of the Raman reporter PPY of the nanotags in MTS incubated with (A) Ab nanotags and (B) cAb nanotags for 4 hours in y = 50 µm increments. All spectra were taken using an x = 10 µm, z = 50 µm step size, a 1 s integration time and 100% laser power of 20 mW and a 785 nm laser excitation. All measurements were taken on n = 3 MTS.

To ascertain if the incubation parameters (incubation time and nanotag concentration) were impacting the observed distribution of nanotags within the MTS, a series of measurements were performed across a range of parameters. Specifically, incubation times of 2, 4, 6, 8, and 24 hours, along with nanotag concentrations of 32 and 128 pM were evaluated (Fig. S7). For these experiments, both Ab and cAb nanotags were studied. Once again, a series of 2D XZ SERS maps were taken across the MTS. The 4-hour time point was chosen as it was deemed that 2 hours was an insufficient length of time for the nanotags to reside within the MTS due to the low SERS signal obtained. A time point of 24 hours was used to determine whether the nanotags would either penetrate further into the MTS, or if over time, the nanotags were removed from the MTS. When the results obtained after 4 hours of incubation are compared to those of the 24-hour incubation (Fig. S7Ai and Aiii, and S7Bi and Biii), it is apparent that neither effect occurred. Even after 24 hours, the nanotags still only appear to be localised to the outer proliferating layers of the MTS. Furthermore, a final nanotag concentration of 128 pM was tried to determine if the nanotag concentration was affecting the penetration depth of the nanotags into the MTS compared to the 32 pM previously tested (Fig. S7Aii and Aiv, and S7Bii and Biv).

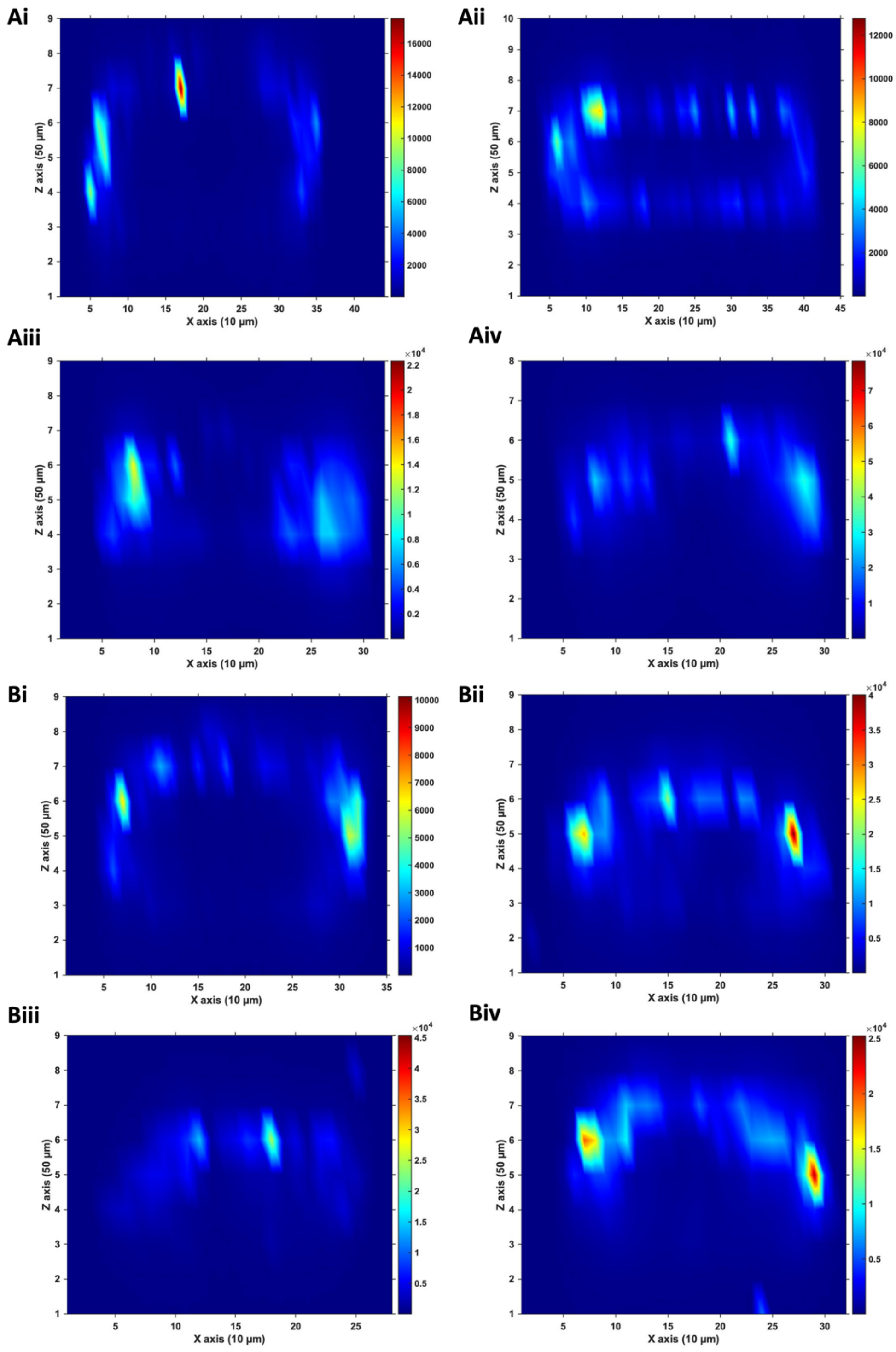

Fig. S7 2D XZ SERS intensity maps at 956 cm^{-1} corresponding to the main intensity peak of the Raman reporter PPY of the nanotags inside MTS incubated with (A) Ab nanotags and (B) cAb nanotags for time and concentration comparisons. i) 32 pM 4-hour incubation, ii) 128 pM 4-hour incubation, iii) 32 pM for 24 hours and iv) 128 pM 24 hours. All spectra were taken using an *x* = 10 µm, *z* = 50 µm step size, a 1 s integration time and 100% laser power (20 mW), with a 785 nm laser excitation. All measurements were taken on n = 3 MTS.

References

1 J. Turkevich, P. C. Stevenson and J. Hillier, *Discuss. Faraday Soc.*, 1951, **11**, 55–75.
