# Supplementary figures and images for: Evaluating nanoparticle localisation in glioblastoma multicellular tumour spheroids by surface enhanced Raman scattering
Source: Analyst. 2023 Jun 16;148(14):3247–56. doi: 10.1039/d3an00751k (PMC10332387; doi:10.1039/d3an00751k)

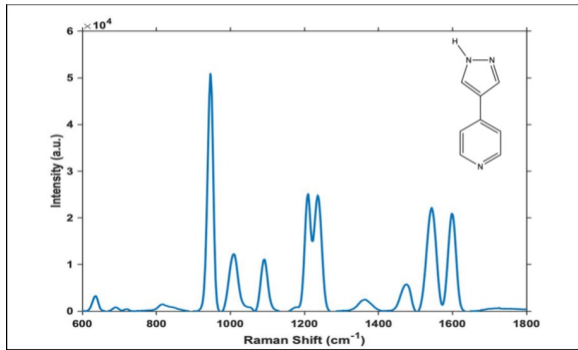

Supplement: AN-148-D3AN00751K-s003 [file AN-148-D3AN00751K-s003.pdf]

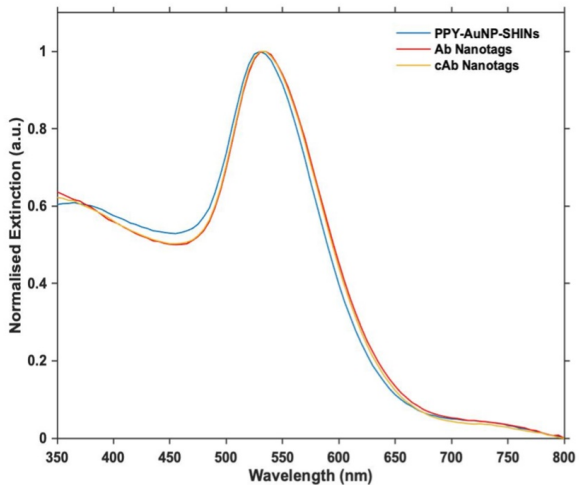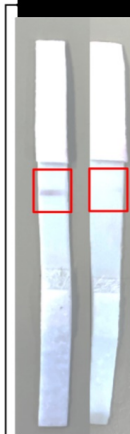

Supplement: AN-148-D3AN00751K-s004 [file AN-148-D3AN00751K-s004.pdf]

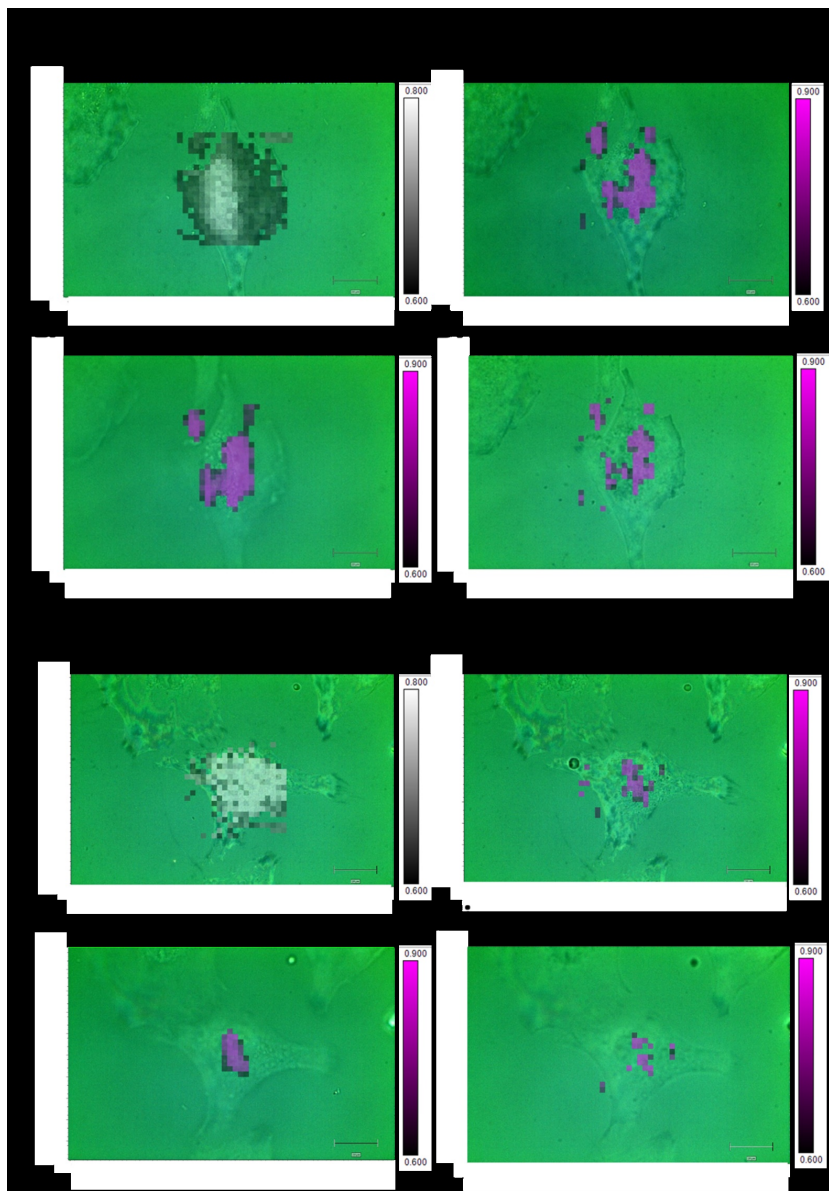

Supplement: AN-148-D3AN00751K-s005 [file AN-148-D3AN00751K-s005.pdf]

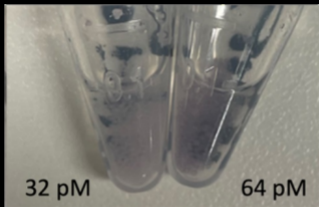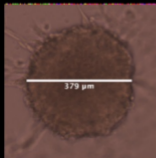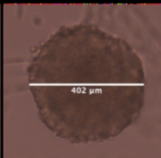

Supplement: AN-148-D3AN00751K-s006 [file AN-148-D3AN00751K-s006.pdf]

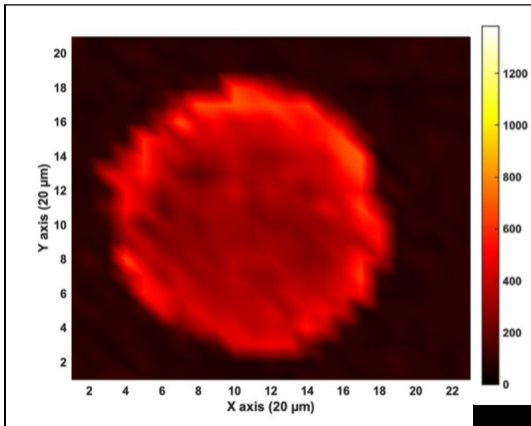

Supplement: AN-148-D3AN00751K-s007 [file AN-148-D3AN00751K-s007.pdf]

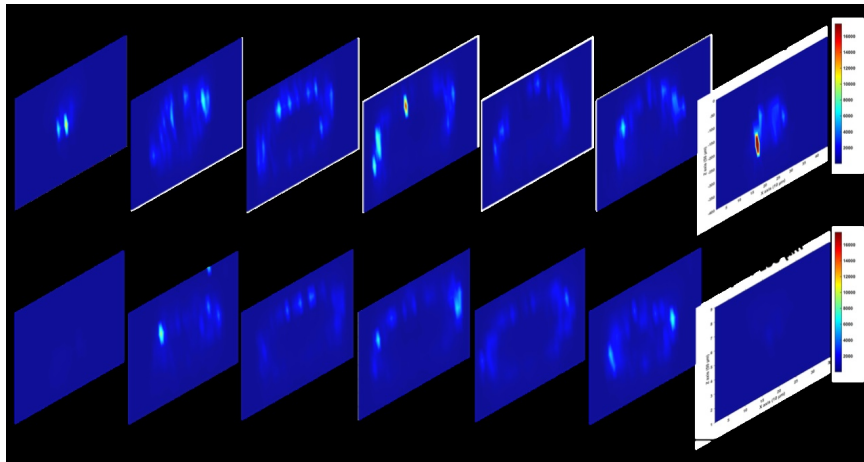

Supplement: AN-148-D3AN00751K-s008 [file AN-148-D3AN00751K-s008.pdf]

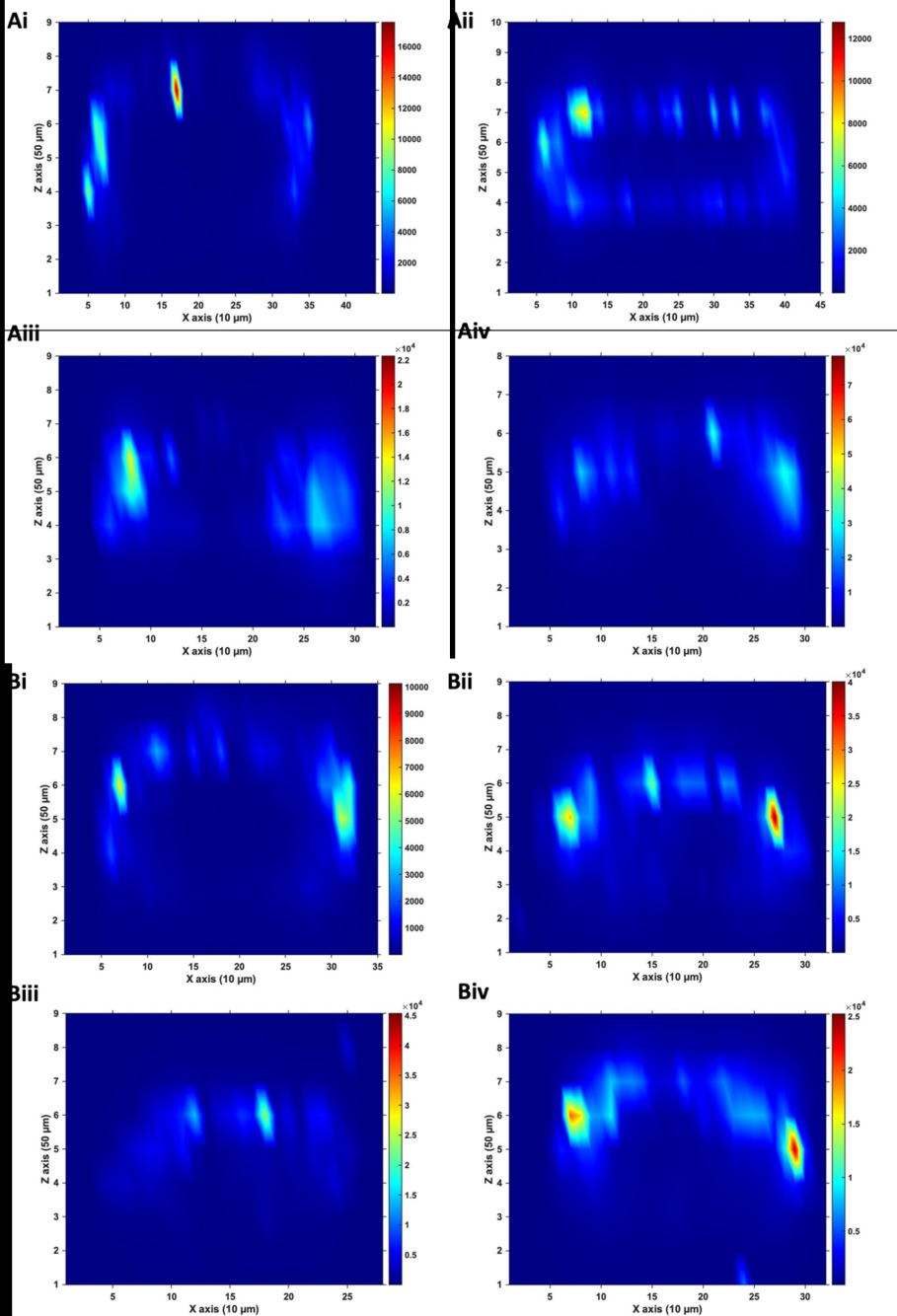

Supplement: AN-148-D3AN00751K-s009 [file AN-148-D3AN00751K-s009.pdf]
